# Mapping the evolution and impact of ketogenic diet research on diabetes management: a comprehensive bibliometric analysis from 2005 to 2024

**DOI:** 10.3389/fnut.2024.1485642

**Published:** 2024-10-15

**Authors:** Zonghuai Li, Anxia Li, Pingping Liu, Bo Zhang, Yuanyuan Yan

**Affiliations:** ^1^Scientific Research Center, Guilin Medical University, Guilin, China; ^2^Department of Pharmacy, Sanya Central Hospital (The Third People's Hospital of Hainan Province), Sanya, Hainan, China

**Keywords:** ketogenic diet, diabetes, bibliometrics, insulin resistance, gut microbiome

## Abstract

**Objective:**

The ketogenic diet (KD) has been explored for diabetes management; however, a quantitative synthesis of its specific effects on diabetes has not yet been conducted. This study aims to examine the current status and research hotspots of KD in diabetes management from 2005 to 2024, providing a reference for future research.

**Methods:**

We retrieved articles published between 2005 and 2024 from the Web of Science database and analyzed them using R software, VOSviewer, and CiteSpace.

**Results:**

This study includes 432 relevant publications. From 2005 to 2024, the volume of literature in this field has shown a steady upward trend, with a notable increase from 2017 to 2021, and a slight decline observed from 2021 to 2023. The United States is the leading country in terms of the number of publications, followed by China, Australia, and Canada. The United States not only leads in publication volume but also maintains a broader international collaboration network. *Nutrients* and the *American Journal of Clinical Nutrition* are the most frequently published and cited journals. Current research hotspots primarily focus on the impact of KD on blood glucose control, insulin resistance, and lipid metabolism in diabetic patients. Mechanistic studies on KD in diabetes management concentrate on aspects such as the “regulation of genes by β-hydroxybutyrate,” “anti-inflammatory effects,” and “oxidative stress.” The role of the gut microbiome is also emerging as an important research area. Currently, exploring the application of KD in managing different age groups and types of diabetes has become a significant research trend.

**Conclusion:**

As an emerging dietary intervention, KD is gradually attracting widespread attention from researchers around the world and is expected to become a major research focus in the future for diabetes management and control. This paper provides a systematic review and analysis of the current research status and hotspots of KD in diabetes management, offering important references and insights for future research in related fields.

## 1 Introduction

Diabetes is a group of metabolic diseases caused by insulin secretion defects or insulin action disorders, primarily characterized by chronic hyperglycemia (1). The pathological mechanisms include impaired pancreatic β-cell function and insulin resistance (2). Hyperglycemia can trigger acute complications such as ketoacidosis and hyperosmolar coma and lead to long-term chronic complications such as cardiovascular disease, neuropathy, nephropathy, and retinopathy (3, 4). Additionally, diabetes is associated with an increased risk of various cancers (5). In recent years, the prevalence of diabetes has rapidly increased. According to the International Diabetes Federation, the global number of diabetes patients has surpassed 460 million and is projected to reach 700 million by 2045 (6). Both developed and developing countries are severely affected by diabetes, particularly due to lifestyle westernization, population aging, and obesity prevalence (7). The burden of diabetes is particularly heavy, significantly impacting patients' quality of life and imposing substantial social and economic costs. Current diabetes treatments include lifestyle interventions, oral hypoglycemic agents, and insulin therapy (8). Lifestyle interventions focus on diet control and increased physical activity (9). Common medications include metformin, sulfonylureas, GLP-1 receptor agonists, and SGLT2 inhibitors (10). Despite these treatments, many patients fail to achieve optimal glycemic control, and medications may have adverse effects. Thus, finding new treatment approaches has become a research focus.

The ketogenic diet (KD) induces ketosis by providing a high-fat, low-carbohydrate, and moderate-protein dietary pattern, replacing glucose with ketone bodies as the primary energy source (11). By significantly reducing carbohydrate intake, it lowers insulin levels, increases fat oxidation, and promotes ketone body production, physiologically mimicking fasting (12). Initially used to treat refractory epilepsy, KD has recently garnered attention for its potential in weight loss and metabolic disease management (13, 14). Several studies have found that the ketogenic diet can modulate the expression of specific microRNAs. For instance, under the ketogenic dietary condition, changes in the levels of miR-Let-7b-5p, miR-143-3p, and miR-504-5p have been observed. These microRNAs are associated with the regulation of mTOR and PPARs signaling pathways (15), which play a critical role in lipid metabolism and contribute significantly to weight reduction in patients (16). HOMA is an index used to quantitatively assess the effects of insulin resistance and β-cell dysfunction on fasting hyperglycemia (17). In one animal study, the ketogenic diet improved insulin sensitivity through the modulation of miRNAs such as miR-205 and miR-411, leading to weight loss and effectively regulating the HOMA index (18). Studies show that KD can significantly lower blood glucose and glycated hemoglobin levels in diabetic patients, improve insulin sensitivity, and aid in weight control (19, 20). Some studies have also shown indicates that KD can improve lipid profiles and reduce cardiovascular risk (21). Further studies indicate that the ketogenic diet markedly improves symptoms in migraine patients, significantly reducing pain (22). It also demonstrates beneficial effects in epilepsy control and has neuroprotective properties (23). For rare conditions like GLUT1 deficiency syndrome, KD has been widely recognized as the gold standard for treatment (24). By dramatically reducing carbohydrate intake and increasing fat consumption, KD enhances fat oxidation and reduces body weight, significantly alleviating symptoms of lipoedema (25). Simultaneously, this dietary pattern inhibits tumor cells from obtaining energy from the microenvironment, showing potential in cancer treatment. These findings collectively suggest that the ketogenic diet is a multifaceted dietary strategy with several health benefits. However, the long-term safety and feasibility of KD in diabetes management remain controversial, necessitating further research to validate its long-term effects and potential risks. For example, prolonged adherence to the ketogenic diet may increase levels of total cholesterol, low-density lipoprotein (LDL), and triglycerides in the body, consequently elevating the risk of cardiovascular diseases such as atherosclerosis (26, 27). Due to the unique nature of the ketogenic diet, individuals may also experience nutritional deficiencies, such as insufficient intake of vitamins A, D, or calcium, which could lead to conditions like night blindness or an increased risk of fractures. Additional side effects may include muscle weakness and arrhythmias (28). Another potential risk is the development of acute kidney injury. In one clinical case, a patient who strictly followed the ketogenic diet and took herbal supplements containing *Uncaria tomentosa* developed acute interstitial nephritis (AIN). The patient's kidney function improved significantly after discontinuing the supplement and receiving corticosteroid treatment (29). Regrettably, the lack of hotspot and frontier analyses in this research field hinders researchers from quickly and accurately identifying future research directions.

Bibliometrics, as a scientific quantitative analysis tool, reveals development trends, hot topics, and frontiers in specific research fields through the statistical analysis of scientific literature (30). For research on KD in diabetes management, bibliometric analysis not only helps systematically review the current research status but also identifies research gaps and future directions, providing reference and guidance for subsequent basic research and clinical applications. Therefore, this study will apply bibliometric methods to comprehensively analyze the research on KD in diabetes treatment, aiming to provide valuable references and insights for scholars and clinicians in the field and promote further development in this area.

## 2 Materials and methods

### 2.1 Data collection

The data we collected were sourced from Web of Science Core Collection (WoSCC; Guangxi Medical University Purchase Edition) on July 11, 2024. We employed the following search formula: ((((TS=(“ketogenic diet”)) AND TS=(diabetes)) AND PY=(2005–2024)) AND DT=(Article OR Review)) AND LA=(English). After eliminating irrelevant documents, a total of 432 papers (excluding duplicates) were identified. The retrieved papers were saved in plain text format and exported as complete records, including cited references.

### 2.2 Data analysis

In this study, we utilized the previously established research methodology (31). For the analysis of annual publications, Origin 2018 was employed. Additionally, R software (version 3.6.3) with the bibliometrix package (version 4.0, http://www.bibliometrix.org) (32), VOSviewer (version 1.6.17) (33), and CiteSpace (version 6.1.4) (34) were utilized. To ensure data accuracy and reliability, two different authors independently performed data extraction and analysis management.

The Bibliometrix software enabled the visual analysis and mapping of scientific knowledge. VOSviewer was employed to visualize country and institutional co-authorship networks, source co-citation analysis, and keyword co-occurrence. In the co-authorship network analysis, parameters were set as follows: minimum number of documents for a country or institution ≥4. For co-citation analysis, the parameter was set as a minimum number of citations for a source ≥50. In keyword co-occurrence analysis, parameters included a minimum number of occurrences of a keyword ≥10, with exclusions of keywords such as “ketogenic diet,” “diabetes” and synonyms of above words. The journal impact factors (IFs) were retrieved from Journal Citation Reports (JCR) in 2023.

## 3 Results

### 3.1 Overview of selected studies on KD in diabetes

**Figure 1:**
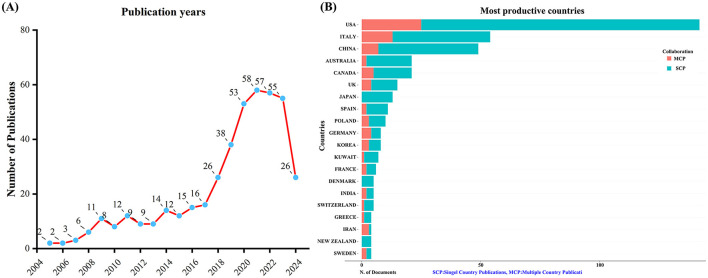
Trends in annual publication outputs on KD in diabetes management from 2005 to 2024. **(A)** Trends of annual publication outputs. **(B)** Distribution of corresponding authors' countries and cooperation.

A total of 432 unique records were obtained from WoSCC after removing duplicates. Between 2005 and 2024, the number of publications concerning KD and diabetes has shown a steady increase, as depicted in Figure 1A. This upward trajectory indicates growing research interest in exploring the connection between KD and diabetes.

**Figure 2:**
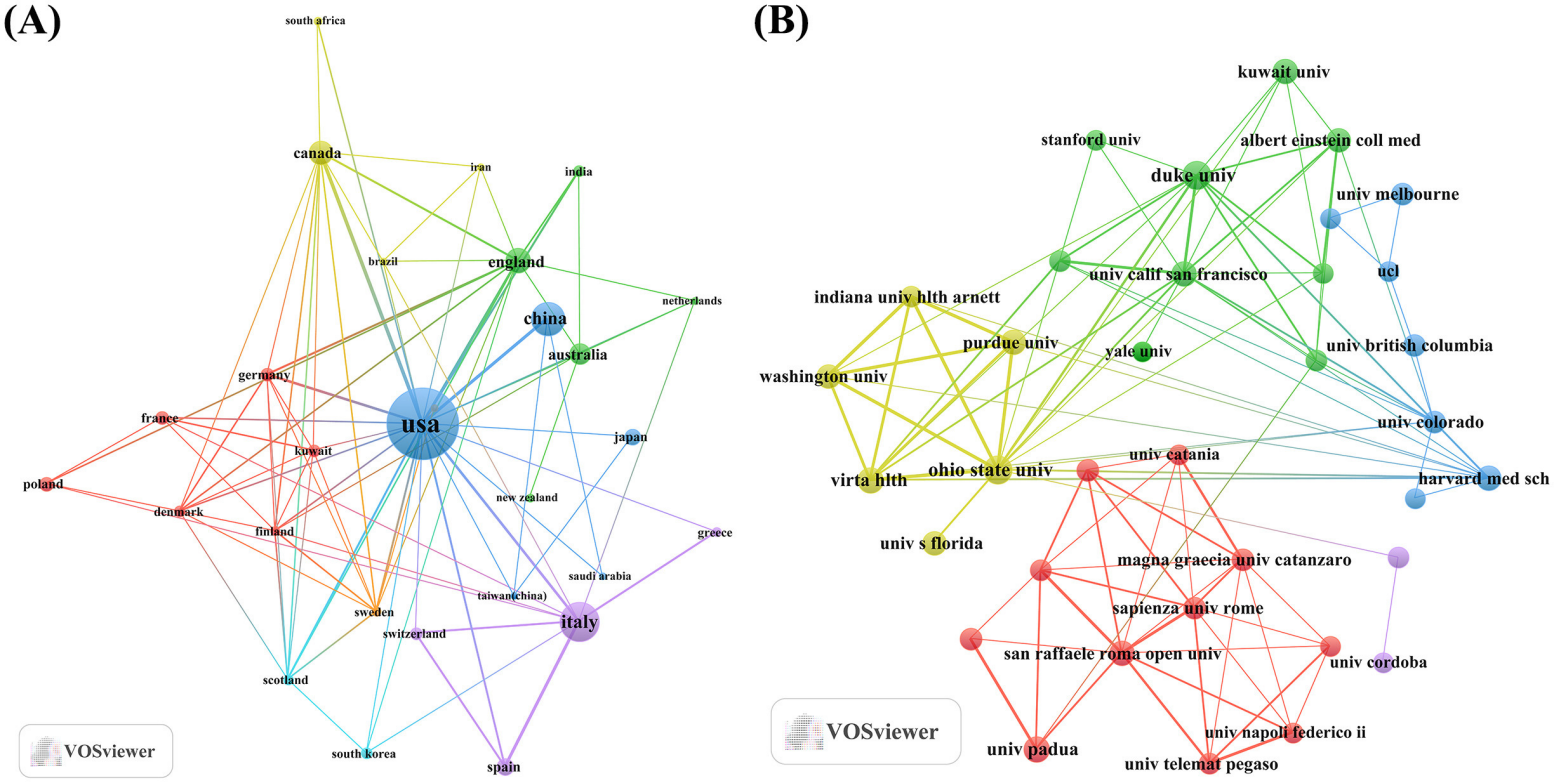
Map of countries/regions and institutions involved in KD research in diabetes management from 2005 to 2024. **(A)** Map of cooperation between different countries. **(B)** Map of cooperation between different institutions.

An analysis of the corresponding authors' countries indicated that USA (*n* = 142) was the leading publisher, followed by the Italy (*n* = 54), China (*n* = 49), Australia (*n* = 21), and Canada (*n* = 21). Moreover, 17.6% of publications from USA and 24.1% from Italy involved multi-country collaborations (MCPs) as shown in Figure 1B and detailed in Table 1. Notably, the USA not only leads in the number of publications but also maintains a broader network of international partnerships, as shown in Figure 2. These data indicate that USA researchers place greater emphasis on studying the effects of KD on diabetes. This may be related to the unique context of the USA, such as dietary habits that are often high in sugar and carbohydrates, which are primary contributors to obesity and diabetes.

**Table 1:** Most relevant countries by corresponding authors.

**Country**	**Articles**	**SCP**	**MCP**	**Freq**	**MCP_Ratio**
USA	142	117	25	0.329	0.176
Italy	54	41	13	0.125	0.241
China	49	42	7	0.113	0.143
Australia	21	19	2	0.049	0.095
Canada	21	16	5	0.049	0.238
United Kingdom	15	11	4	0.035	0.267
Japan	13	13	0	0.03	0
Spain	11	9	2	0.025	0.182
Poland	10	7	3	0.023	0.3
Germany	8	4	4	0.019	0.5
Korea	8	5	3	0.019	0.375
Kuwait	7	6	1	0.016	0.143
France	6	4	2	0.014	0.333
Denmark	5	5	0	0.012	0
India	5	3	2	0.012	0.4
Switzerland	5	4	1	0.012	0.2
Greece	4	3	1	0.009	0.25
Iran	4	1	3	0.009	0.75
New Zealand	4	4	0	0.009	0
Sweden	4	2	2	0.009	0.5

MCP, Multiple country publication; SCP, Single country publication.

### 3.2 Journal analysis and visualization

For examining the journals with the highest publication and citation contributions in the field of KD and diabetes, we utilized the Bibliometrix package in R software. Graphical representations were created using the ggplot2 package. Furthermore, co-cited journal analysis was conducted using VOSviewer.

**Figure 3:**
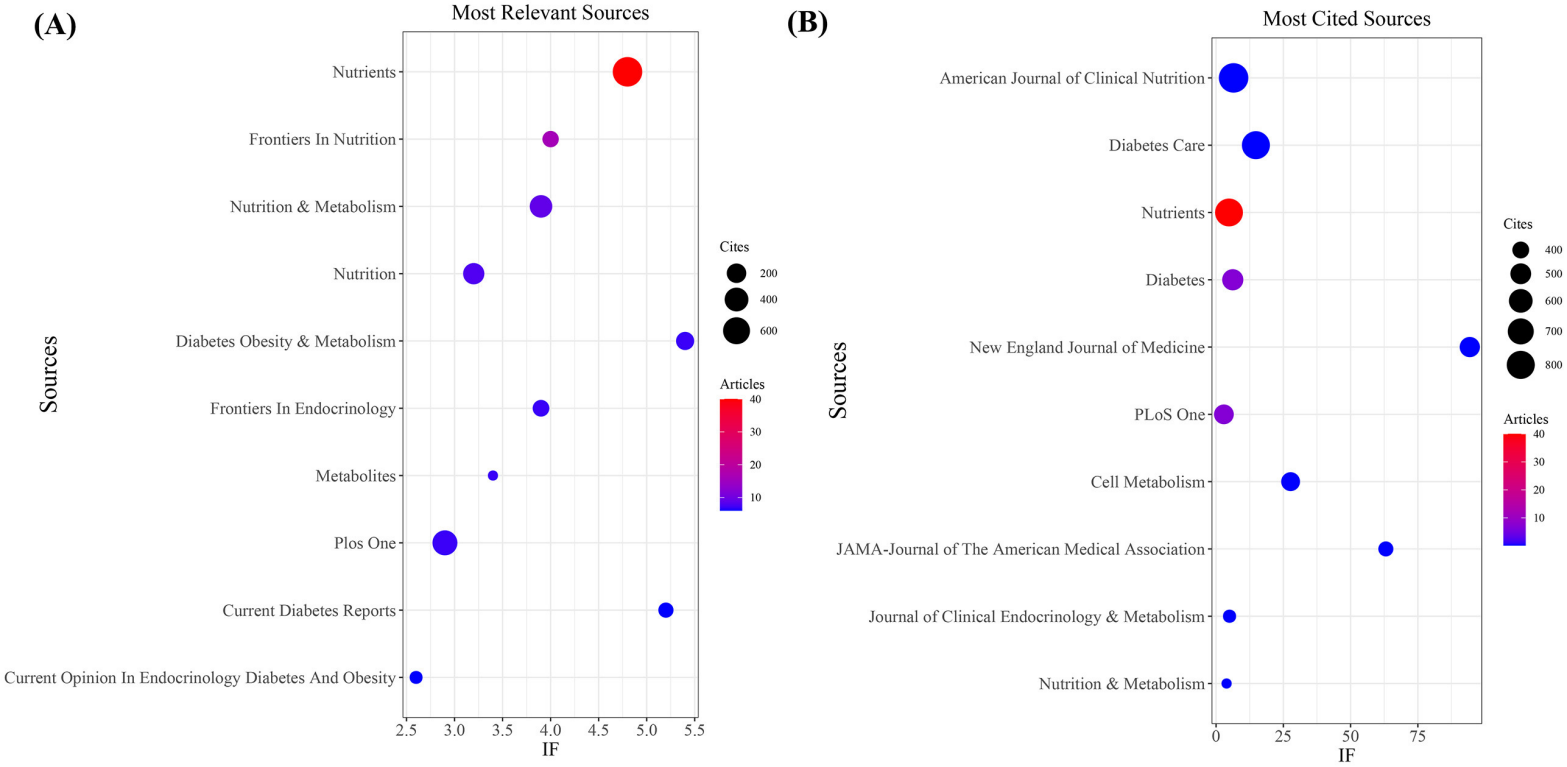
Journal with the largest number of articles published and the journal with the largest number of citations. **(A)** Journal with the largest number of articles published. **(B)** Journals with the largest number of citations.

**Figure 4:**
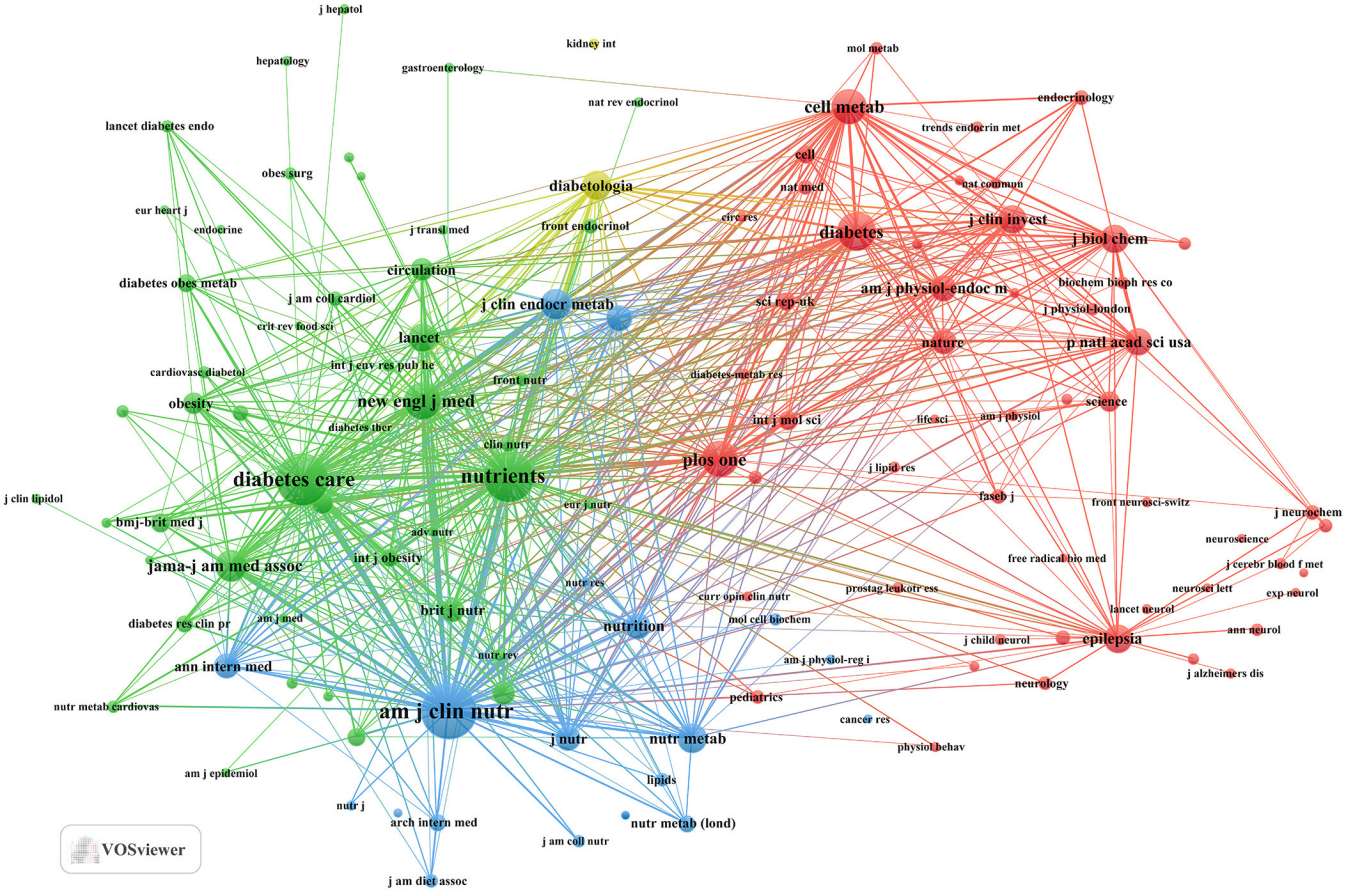
Co-cited journals related to KD in diabetes management.

Our investigation yielded a total of 432 documents dispersed across 230 scholarly journals (see Supplementary material 1 for detailed information). As illustrated in Table 2 and depicted in Figure 3A, the *Nutrients* (*n* = 40, IF = 4.8) emerged as the leading publisher, followed by *Frontiers in Nutrition* (*n* = 16, IF = 4), *Nutrition and Metabolism* (*n* = 9, IF = 3.9), *Nutrition* (*n* = 8, IF = 3.2), and *Diabetes Obesity and Metabolism* (*n* = 7, IF = 5.4). Table 3 and Figure 3B showcase the most frequently cited journals, including *American Journal of Clinical Nutrition* (*n* = 877, IF = 6.5), *Diabetes Care* (*n* = 797, IF = 14.8), *Nutrients* (*n* = 789, IF = 4.8), *Diabetes* (*n* = 512, IF = 6.2), and *New England Journal of Medicine* (*n* = 483, IF = 94.3). Notably, the co-cited journals map in Figure 4 demonstrates that *Nutrients, Diabetes Care*, and *American Journal of Clinical Nutrition* act as central collaboration hubs. These findings collectively underscore the influential role of the *Nutrients* in the field of KD in diabetes.

**Table 2:** Top 10 journals with the most published.

**Sources**	**Documents**	**IF (2023)**	**Cites**
Nutrients	40	4.8	789
Frontiers In Nutrition	16	4	99
Nutrition and Metabolism	9	3.9	335
Nutrition	8	3.2	275
Diabetes Obesity and Metabolism	7	5.4	151
Frontiers In Endocrinology	7	3.9	108
Metabolites	7	3.4	19
PLoS One	7	2.9	469
Current Diabetes Reports	6	5.2	72
Current Opinion In Endocrinology Diabetes And Obesity	6	2.6	35

**Table 3:** Top 10 journals with the most cited.

**Sources**	**Cites**	**IF (2023)**	**Documents**
American Journal of Clinical Nutrition	877	6.5	0
Diabetes Care	797	14.8	0
Nutrients	789	4.8	40
Diabetes	512	6.2	7
New England Journal of Medicine	483	94.3	0
PLoS One	469	2.9	7
Cell Metabolism	446	27.7	0
JAMA-Journal of The American Medical Association	370	63.1	0
Journal of Clinical Endocrinology and Metabolism	348	5	0
Nutrition and Metabolism	335	3.9	0

### 3.3 Citation bursts

**Figure 5:**
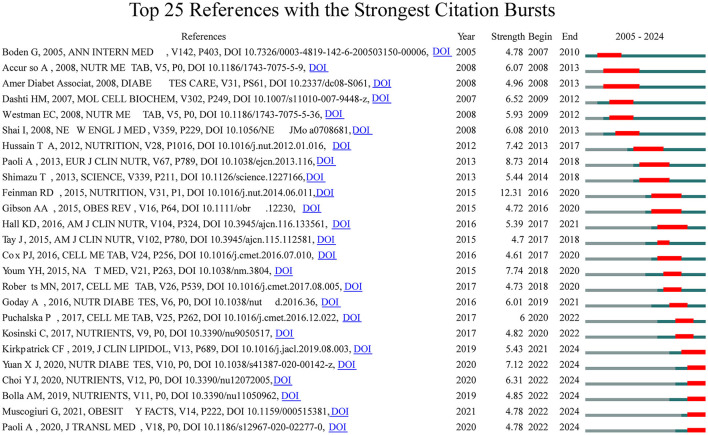
Top 25 references with the strongest citation bursts in KD for diabetes management.

To delve deeper into the exploration of the forefront and focal areas of KD in diabetes, we utilized CiteSpace to identify the top 25 most significant citation bursts related to KD in diabetes (refer to Figure 5). The titles of these citations, along with their respective DOIs, are listed in Supplementary material 2. Remarkably, the three citations exhibiting the most pronounced citation bursts were: (1) “Dietary carbohydrate restriction as the first approach in diabetes management: critical review and evidence base (strength: 12.31);” (2) “Beyond weight loss: a review of the therapeutic uses of very-low-carbohydrate (ketogenic) diets (strength: 8.73);” (3) “The ketone metabolite β-hydroxybutyrate blocks NLRP3 inflammasome–mediated inflammatory disease (strength: 7.74).” Furthermore, the titles of the three most cutting-edge citation bursts were: (1) “Effects of a ketogenic diet in overweight women with polycystic ovary syndrome;” (2) “European guidelines for obesity management in adults with a very low-calorie ketogenic diet: a systematic review and meta-analysis;” (3) “Low-carb and ketogenic diets in type 1 and type 2 diabetes.”

Overall, through the most cited references and reference burst analysis, we have identified two key areas of focus within the field of KD in diabetes: 1. Metabolic effects of KD on diabetes patients, including glycemic control, insulin resistance, and lipid metabolism; 2. Mechanistic studies of KD, including the multi-dimensional roles of ketone bodies (such as β-hydroxybutyrate) in metabolism, signaling, and therapeutics.

### 3.4 Keyword clusters and evolution of themes

Keyword clusters are essential for rapidly grasping the main research themes and directions in a particular area. In our study, VOSviewer was used to identify 2,145 keywords. Table 4 displays the top 20 keywords, each occurring more than 27 times, highlighting the prominent research focuses. The most frequently appearing keyword was “weight loss” (*n* = 131), followed by “obesity” (*n* = 127), “insulin-resistance” (*n* = 108), “ketone-bodies” (*n* = 62), “beta-hydroxybutyrate” (*n* = 56), and “metabolism” (*n* = 56).

**Table 4:** The top 20 keywords.

**Rank**	**Keywords**	**Count**
1	Weight loss	131
2	Obesity	127
3	Insulin-resistance	108
4	Ketone-bodies	62
5	Beta-hydroxybutyrate	56
6	Metabolism	56
7	Cardiovascular risk-factors	48
8	Oxidative stress	42
9	Metabolic syndrome	41
10	Management	40
11	Ketosis	37
12	Glucose	34
13	Insulin	34
14	Glycemic control	33
15	Epilepsy	32
16	Cardiovascular disease	31
17	Inflammation	30
18	Nutrition	30
19	Body-composition	29
20	Mediterranean diet	27

**Figure 6:**
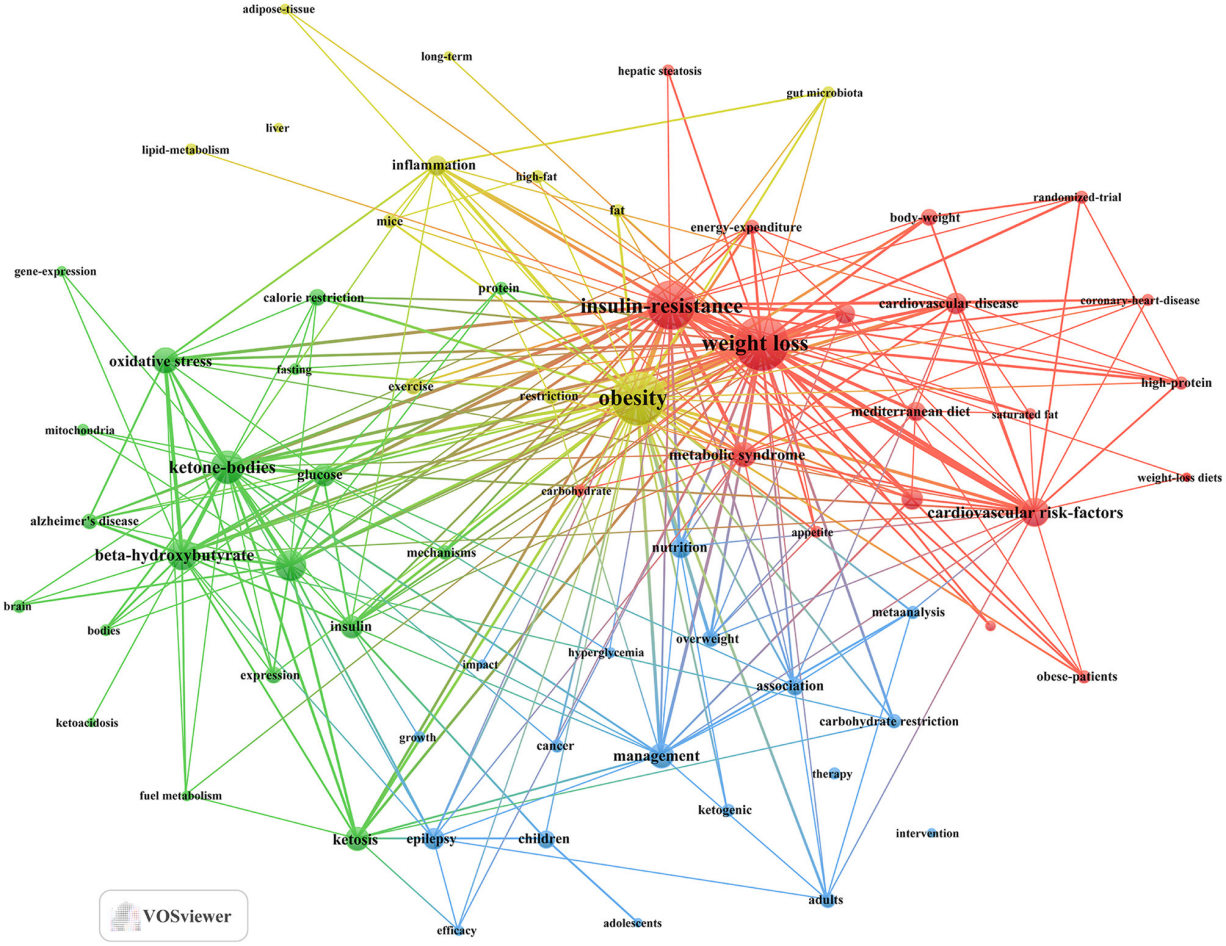
Keyword co-occurrence map of publications on KD in diabetes management.

Through cluster analysis, we observe four different colored clusters in Figure 6. (1) Effects of the ketogenic diet on diabetes and related metabolic syndrome (red dots), there are 20 keywords, including energy-expenditure, glycemic control, insulin-resistance, metabolic syndrome, weight loss, and so on. (2) Metabolic and physiological mechanisms of the ketogenic diet in diabetes management (green dots), there are 19 keywords, including ketone-bodies, mechanisms, metabolism, mitochondria, oxidative stress, and so on. (3) Efficacy and applications of the ketogenic diet in different populations (blue dots), there are 18 keywords, including adults, children, hyperglycemia, intervention, therapy, and so on. (4) Long-term effects of the ketogenic diet on diabetes-related lipid metabolism and inflammation (yellow dot), there are 12 keywords, including inflammation, lipid-metabolism, gut microbiota, long-term, obesity, and so on. All keywords contained in the four clusters can be found in Supplementary material 3.

**Figure 7:**
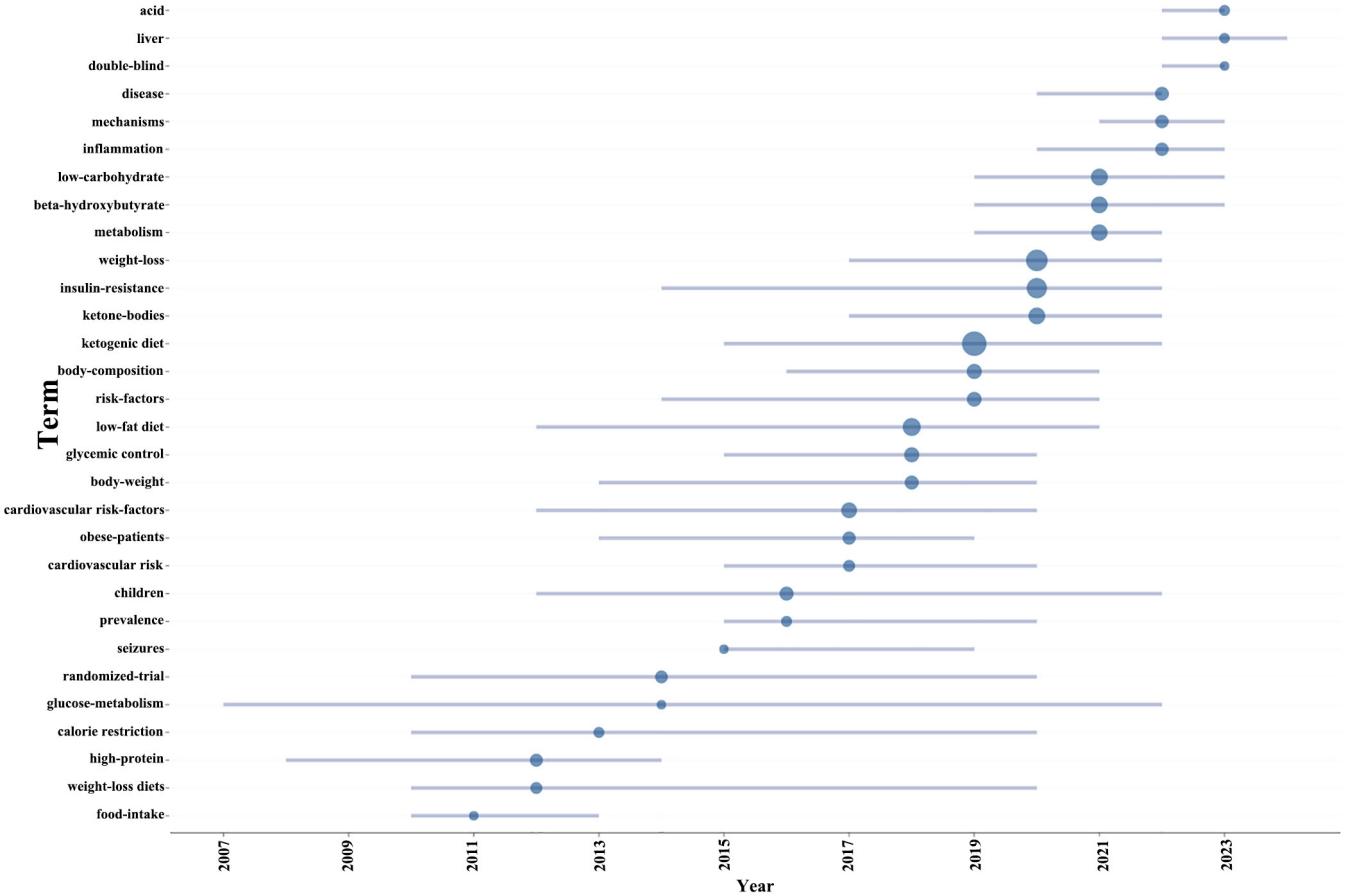
Trend topics on KD in diabetes management.

Additionally, in order to project forthcoming trends within this domain, we utilized the bibliometrix toolkit within the R programming environment to construct a dynamic thematic progression chart (Figure 7). The period from 2005 to 2013 was characterized by a predominant focus on the pathophysiological mechanisms of diabetes, encompassing insulin resistance and metabolic disorders, alongside the exploration of diagnostic approaches and disease classification. Transitioning into the years 2013 to 2015, there was a discernible shift toward the examination of the ketogenic diet's potential impact on diabetes management, with a particular emphasis on weight loss and body composition modulation, as well as the influence of low-carbohydrate diets on metabolic pathways. From 2015 to 2021, the research community expanded its scope to include the effects of ketogenic diets on inflammation and cardiovascular risk factors, while also investigating the implications of glycemic control and the role of high-protein, weight-loss diets. In the most recent phase, spanning 2021 to 2023, the adoption of rigorous scientific methodologies, such as randomized trials and double-blind studies, became prominent, enhancing the validity of research findings. Additionally, the impact of low-fat diets and calorie restriction on diabetes and weight management garnered increased attention. Our thematic evolution map suggests that future research is poised to delve deeper into the risk factors associated with diabetes, especially in pediatric populations and the prevalence of the disease. Furthermore, obtaining more comprehensive and reliable clinical data will be crucial for advancing research on the ketogenic diet's impact on diabetes.

### 3.5 Comprehensive analysis of hotspots

In conclusion, our extensive analysis, which includes citation bursts, keyword frequency analysis, keyword clustering, and thematic evolution, has identified emerging research hotspots in the intersection of KD and diabetes. Our findings reveal that the research hotspots in this field are primarily focused on three key directions. (1) Metabolic effects of KD in diabetic patients: impacts on blood glucose control, insulin resistance, and lipid metabolism. (2) Mechanisms of KD in diabetes improvement: roles of ketone bodies in metabolic and signaling pathways. (3) Application of KD in various populations: efficacy across different age groups and types of diabetes, supported by clinical evidence.

## 4 Discussion

### 4.1 General information

In this study, we collected a comprehensive corpus of 432 publications covering the period from 2004 to 2024. The results indicate a steady increase in the number of publications on the KD and diabetes from 2004 to 2024. This upward trend suggests a growing interest among researchers in exploring the relationship between KD and diabetes. However, it is noteworthy that there has been a decline in the number of publications in 2022 and 2023 compared to 2021. This decline may be attributed to the following four reasons: (1) Conceptual Ambiguity: The definition of the KD is not uniform across different studies, particularly regarding standards and methods for calculating carbohydrate intake. Variations in carbohydrate intake standards (e.g., net carbohydrates vs. total carbohydrates) and whether other factors (such as protein intake) are considered can affect the comparability and consistency of study results. The lack of a clear definition of KD poses significant challenges for researchers (11). (2) Participant Adherence and Long-Term Sustainability: KD is a highly restrictive dietary pattern requiring participants to strictly control carbohydrate intake. Many studies have found that participants struggle to adhere to this diet over the long term, which affects the reliability of study results and increases the difficulty of follow-up and data collection (35, 36). (3) High Restrictiveness of KD: KD imposes strict limitations on daily food intake, and long-term adherence may lead to side effects such as visceral damage, which can reduce patient adherence (37, 38). Consequently, researchers face increased challenges in exploring this area. (4) Shift in Research Interest: Over time, researchers and clinicians have shown increased interest in other dietary interventions, such as the Mediterranean diet and low-calorie diets. These methods have demonstrated better outcomes in some studies for diabetes management (39). As a result, research interest has gradually shifted from KD to these alternative dietary approaches.

In the field of research on the relationship between KD and diabetes, the United States is the country with the highest number of published articles. This trend may be related to the unique dietary environment in the U.S., where diets typically include high levels of carbohydrates and sugary foods, potentially leading to a higher prevalence of diabetes and thus a greater need to explore new dietary habits. The 432 publications are distributed across 230 journals, with prominent journals such as *Nutrients, Frontiers in Nutrition*, and *Nutrition and Metabolism* contributing a substantial number of articles. Notably, *Nutrients* has emerged as a major research focus, publishing a significant volume of articles and receiving a considerable number of citations. Its prominent position highlights *Nutrients* as a key publication in the field of KD and diabetes research, confirming its role as a primary channel for disseminating research findings in this area.

### 4.2 Hotspots and development trends

As mentioned above, through a comprehensive analysis of literature clustering, keyword frequency analysis, keyword clustering, and topic evolution, we identified potential research hotspots regarding the impact of KD on diabetes. The results show that the research frontiers and hotspots in this field mainly focus on three aspects. First, the metabolic effects of KD on diabetic patients, including its impact on blood glucose control, insulin resistance, and lipid metabolism. Second, the mechanisms by which KD improves diabetes, particularly the role of ketone bodies in metabolic and signaling pathways. Finally, the application of KD in different populations, including its efficacy in various age groups and types of diabetes, with existing clinical evidence supporting these applications.

#### 4.2.1 The metabolic effects of KD on diabetic patients, including its impact on blood glucose control, insulin resistance, and lipid metabolism

Based on our analysis of the existing literature, the ketogenic diet exerts a substantial regulatory impact on blood glucose control, insulin resistance, and lipid metabolism. One significant characteristic of KD is its low carbohydrate intake, which reduces the availability of glucose as an energy source (40, 41). HbA1c, a commonly used marker for blood glucose control, reflects the average blood glucose levels over the past 3 months (42). Research indicates that KD can effectively improve blood glucose fluctuations in individuals with Type 1 diabetes and lower HbA1c levels (43, 44). Additionally, low carbohydrate intake further shifts the body's energy supply from glucose metabolism to lipid metabolism, inducing a new metabolic pathway (45). This metabolic pathway can enhance the efficiency of fat metabolism, particularly visceral fat. However, increased visceral fat may also lead to further insulin resistance (46, 47). Importantly, KD also affects the composition of lipids in the body. It can modestly lower low-density lipoprotein cholesterol (LDL-C), known as “bad” cholesterol (48), while modestly increasing high-density lipoprotein cholesterol (HDL-C), known as “good” cholesterol (49, 50).

#### 4.2.2 Mechanisms by which KD improves diabetes, particularly the role of ketone bodies in metabolic and signaling pathways

Through the analysis of keywords and related information, we identified that the primary mechanisms by which the ketogenic diet affects diabetic patients include the regulation of β-hydroxybutyrate-related genes, anti-inflammatory effects, and oxidative stress. During the ketogenic diet, oxaloacetate rapidly participates in gluconeogenesis; however, as the concentration of ketone bodies in the body increases, the production of hepatic glycogen gradually declines. Therefore, the ketogenic diet can modulate the level of gluconeogenesis to a certain extent, effectively preventing excessive blood glucose levels and achieving good glycemic control (51). In the pathway of insulin resistance, the effector protein GLUT4 is closely related to the fatty acid metabolism-associated protein HADH1 (52). GLUT4 translocates from storage vesicles to the cell membrane under insulin stimulation, subsequently acting as a glucose transporter to facilitate glucose entry into the cells, thereby lowering blood glucose levels. The enhanced expression of HADH1 regulates GLUT4 activity, thereby improving insulin sensitivity (53, 54). β-Hydroxybutyrate (βOHB) is one of the primary ketone bodies in KD (55). Research indicates that βOHB can inhibit the activity of certain histone deacetylases (HDACs) (56). HDACs regulate gene expression by removing acetyl groups from histones (57). When HDACs are inhibited, the expression of brain-derived neurotrophic factor (BDNF) is enhanced, and BDNF expression can reduce oxidative stress and inflammatory responses in the body (56). In addition, HDAC inhibition can reduce the activity of nuclear factor κB (NF-κB) (58). These factors play a key role in the expression of pro-inflammatory cytokines such as TNF-α and IL-1β (59), which are crucial contributors to the inflammatory response (58). Oxidative stress and inflammation induced by diabetes can impair β-cell function, leading to insulin resistance and disrupted blood glucose regulation (60), which in turn causes a range of diabetes-related complications (61, 62). Additionally, a low-carbohydrate diet can improve the gut microbiota through various mechanisms (63). For example, it can increase the abundance of Lactobacillus murinus ASF361, which encodes bile salt hydrolase (BSH). This change leads to elevated levels of tauroursodeoxycholic acid (TDCA) and tauroursodeoxycholic acid (TUDCA) in the serum. These bile acids reduce intestinal energy absorption by inhibiting the expression of intestinal carbonic anhydrase 1 (Car1). This process not only helps with weight loss but may also lower fasting blood glucose levels, thereby positively impacting metabolic diseases like diabetes (60). Through these mechanisms, KD may offer new perspectives for improving metabolic health, particularly in the management and treatment of diabetes.

#### 4.2.3 Application of KD in different populations, including its efficacy for various age groups and types of diabetes, with existing clinical evidence supporting it

Certainly, after analysis, investigating diabetes across various age groups and types is currently a focal point of research in this field. (1) Type 1 Diabetes (T1D) in Adults: Before the discovery of insulin, strict daily control of carbohydrate intake was the only effective treatment for T1D (64). Despite advances in modern medicine with various treatment options for T1D, KD remains an effective method for blood glucose control (65). Numerous studies have shown that KD not only helps reduce blood glucose fluctuations but also lowers HbA1c levels (66, 67). However, maintaining a KD long-term presents a significant challenge for many patients, as most find it difficult to adhere to this diet over time (68). (2) T1D in Children: Many studies indicate that the KD can provide significant benefits for children with T1D in the short term (69). However, regular monitoring of growth and nutritional markers is necessary to ensure these remain within normal ranges (70). This is because the KD may increase the risk of cardiovascular diseases and other health issues in children (71, 72). (3) Type 2 Diabetes (T2D) in Adults: As demonstrated by Goldenberg et al., short-term KD can significantly improve blood glucose control and aid in weight management for T2D patients (36). However, further research is needed to clarify the long-term effects and potential risks of sustained KD. (4) T2D in Children: Research on adolescent T2D patients is still limited. A retrospective study showed that adolescents following a KD experienced short-term diabetes remission and reduced BMI. However, due to participant attrition in the study, the long-term effects may be underestimated (73).

### 4.3 Strengths and limitations

This study provides a comprehensive overview of the general landscape, hotspots, and research trends in a specific field using the WoSCC database as the data source, aiding in a deeper understanding of the field and exploring future research directions. However, this study also has some notable limitations. First, reliance solely on the WoSCC database may result in the omission of some relevant literature, despite its reputation for high quality and broad recognition as an ideal tool for bibliometric analysis (74). Second, this study includes only English-language publications, which may introduce language bias and limit the generalizability of the findings. Additionally, due to the large number of authors with similar names in China, this study did not conduct an in-depth analysis of authors to avoid potential misleading results. Furthermore, analyses conducted using CiteSpace and VOSviewer cannot fully replace systematic searches, and bibliometrics cannot assess the quality of individual studies, as citation metrics are time-dependent, with recent articles possibly having fewer citations primarily due to their publication date (75). Despite these limitations, the conclusions of this study remain highly reliable and provide valuable insights and references for academic research in the field, laying the groundwork for understanding (76). However, the long-term effects of the ketogenic diet on diabetic patients, as well as the potential for other adverse effects, still require further investigation and analysis by additional researchers.

## 5 Conclusion

This study provides an in-depth exploration of the primary research hotspots and cutting-edge trends related to the application of the ketogenic diet in diabetes management. The key findings are summarized as follows:

a. The application of the ketogenic diet in diabetes management has garnered substantial global attention, particularly from researchers in the United States, China, Australia, and Canada. These countries are among the most active in this field, and there is significant international collaboration.
b. Within this research domain, Nutrients and Frontiers in Nutrition are the most active journals. The American Journal of Clinical Nutrition and Diabetes Care are also frequently cited, with Nutrients standing out for both its publication volume and citation frequency, indicating its prominence as a representative journal in this field.
c. Current research hotspots include the impact of the ketogenic diet on glucose control, insulin resistance, and lipid metabolism in diabetic patients.
d. Mechanistic studies on the ketogenic diet for diabetes management are focusing on trends such as “β-hydroxybutyrate-regulated genes,” “anti-inflammatory effects,” and “oxidative stress.” Additionally, the role of the “gut microbiome” is emerging as an important area of interest.
e. Research on the management of various types of diabetes across different age groups with the ketogenic diet is a prominent current trend.

In summary, this study offers valuable insights into the research trends and hotspots related to the ketogenic diet in diabetes management by outlining current research prospects and potential focal areas. The findings not only enhance the understanding of the existing state of research but also establish a robust foundation for future research directions. The impact of the ketogenic diet on microRNA is likely to become a primary focus in upcoming studies, as it may promote effective weight reduction and significant improvements in the HOMA index, thereby providing substantial benefits for diabetic patients. However, the potential adverse effects associated with long-term adherence to the ketogenic diet, such as cardiovascular diseases and nutritional deficiencies, must also be addressed as critical areas of investigation. Furthermore, given that the ketogenic diet may influence insulin sensitivity and fat metabolism differently among individuals, personalized ketogenic dietary approaches could emerge as a significant research direction in diabetes management.

## Data Availability

The original contributions presented in the study are included in the article/Supplementary material, further inquiries can be directed to the corresponding author/s.

## References

[1] Association AD. Diagnosis and classification of diabetes mellitus. Diabet Care. (2014) 37(Suppl.1):S81–90. 10.2337/dc14-S081 24357215

[2] Eizirik DL Pasquali L Cnop M. Pancreatic β-cells in type 1 and type 2 diabetes mellitus: different pathways to failure. Nat Rev Endocrinol. (2020) 16:349–62. 10.1038/s41574-020-0355-7 32398822

[3] Mohajan D Mohajan HK. Hyperglycaemia among diabetes patients: a preventive approach. Innov Sci Technol. (2023) 2:27–33. 10.56397/IST.2023.11.05

[4] Farmaki P Damaskos C Garmpis N Garmpi A Savvanis S Diamantis E. Complications of the type 2 diabetes mellitus. Curr. Cardiol. Rev. (2020) 16:249–51. 10.2174/1573403X1604201229115531 33407062 PMC7903505

[5] Ling S Zaccardi F Issa E Davies MJ Khunti K Brown K. Inequalities in cancer mortality trends in people with type 2 diabetes: 20 year population-based study in England. Diabetologia. (2023) 66:657–73. 10.1007/s00125-022-05854-8 36690836 PMC9947024

[6] Kowluru RA Mohammad G. Epigenetic modifications in diabetes. Metabolism. (2022) 126:154920. 10.1016/j.metabol.2021.154920 34715117 PMC10277168

[7] Lin X Xu Y Pan X Xu J Ding Y Sun X. Global, regional, and national burden and trend of diabetes in 195 countries and territories: an analysis from 1990 to 2025. Sci Rep. (2020) 10:1–11. 10.1038/s41598-020-71908-9 32901098 PMC7478957

[8] Prasathkumar M Becky R Anisha S Dhrisya C Sadhasivam S. Evaluation of hypoglycemic therapeutics and nutritional supplementation for type 2 diabetes mellitus management: an insight on molecular approaches. Biotechnol Lett. (2022) 44:203–38. 10.1007/s10529-022-03232-3 35119572

[9] Zhang Y Yang Y Huang Q Zhang Q Li M Wu Y. The effectiveness of lifestyle interventions for diabetes remission on patients with type 2 diabetes mellitus: a systematic review and meta-analysis. Worldv Evid Based Nurs. (2023) 20:64–78. 10.1111/wvn.12608 36480153

[10] Xie Y Bowe B Xian H Loux T McGill JB Al-Aly Z. Comparative effectiveness of SGLT2 inhibitors, GLP-1 receptor agonists, DPP-4 inhibitors, and sulfonylureas on risk of major adverse cardiovascular events: emulation of a randomised target trial using electronic health records. Lancet Diabet Endocrinol. (2023) 11:644–56. 10.1016/S2213-8587(23)00171-7 37499675

[11] Firman CH Mellor DD Unwin D Brown A. Does a ketogenic diet have a place within diabetes clinical practice? Review of current evidence and controversies. Diabet Ther. (2024) 15:77–97. 10.1007/s13300-023-01492-4 37966583 PMC10786817

[12] Paoli A Cerullo G. Investigating the link between ketogenic diet, NAFLD, mitochondria, and oxidative stress: a narrative review. Antioxidants. (2023) 12:1065. 10.3390/antiox12051065 37237931 PMC10215390

[13] Zarnowska IM. Therapeutic use of the ketogenic diet in refractory epilepsy: what we know and what still needs to be learned. Nutrients. (2020) 12:2616. 10.3390/nu12092616 32867258 PMC7551948

[14] Verrotti A Iapadre G Di Francesco L Zagaroli L Farello G. Diet in the treatment of epilepsy: what we know so far. Nutrients. (2020) 12:2645. 10.3390/nu12092645 32872661 PMC7551815

[15] Cannataro R Perri M Gallelli L Caroleo MC De Sarro G Cione E. Ketogenic diet acts on body remodeling and MicroRNAs expression profile. MicroRNA. (2019) 8:116–26. 10.2174/2211536608666181126093903 30474543

[16] Hernández-Gómez KG Avila-Nava A González-Salazar LE Noriega LG Serralde-Zúñiga AE Guizar-Heredia R. Modulation of MicroRNAs and exosomal MicroRNAs after dietary interventions for obesity and insulin resistance: a narrative review. Metabolites. (2023) 13:1190. 10.3390/metabo13121190 38132872 PMC10745452

[17] Adi N Adi J Lassance-Soares RM Kurlansky P Yu H Webster KA. High protein/fish oil diet prevents hepatic steatosis in NONcNZO10 mice; association with diet/genetics-regulated micro-RNAs. J Diabetes Metab. (2016) 7:676. 10.4172/2155-6156.1000676 28529818 PMC5436721

[18] Nolan JJ Færch K. Estimating insulin sensitivity and beta cell function: perspectives from the modern pandemics of obesity and type 2 diabetes. Diabetologia. (2012) 55:2863–7. 10.1007/s00125-012-2684-0 22911384

[19] Hussain TA Mathew TC Dashti AA Asfar S Al-Zaid N Dashti HM. Effect of low-calorie versus low-carbohydrate ketogenic diet in type 2 diabetes. Nutrition. (2012) 28:1016–21. 10.1016/j.nut.2012.01.016 22673594

[20] Paoli A Bianco A Moro T Mota JF Coelho-Ravagnani CF. The effects of ketogenic diet on insulin sensitivity and weight loss, which came first: the chicken or the egg? Nutrients. (2023) 15:3120. 10.3390/nu15143120 37513538 PMC10385501

[21] Dyńka D Kowalcze K Charuta A Paziewska A. The ketogenic diet and cardiovascular diseases. Nutrients. (2023) 15:3368. 10.3390/nu15153368 37892389 PMC10609625

[22] Nathan J Bailur S Datay K Sharma S Khedekar Kale D. A switch to polyunsaturated fatty acid based ketogenic diet improves seizure control in patients with drug-resistant epilepsy on the mixed fat ketogenic diet: a retrospective open label trial. Cureus. (2019) 11:e6399. 10.7759/cureus.6399 31886101 PMC6919946

[23] Merlino G Tereshko Y Pez S Bello SD Pittino A Di Lorenzo C. Sleep of migraine patients is ameliorated by ketogenic diet, independently of pain control. Sleep Med. (2023) 107:196–201. 10.1016/j.sleep.2023.05.006 37209426

[24] Varesio C Zanaboni MP Pasca L Provenzi L Ferraris C Tagliabue A. Novel insight into GLUT1 deficiency syndrome: screening for emotional and behavioral problems in youths following ketogenic diet. Minerva Pediatr. (2024) 76:189–96. 10.23736/S2724-5276.21.05923-1 33820407

[25] Verde L Camajani E Annunziata G Sojat A Marina LV Colao A. Ketogenic diet: a nutritional therapeutic tool for lipedema? Curr Obes Rep. (2023) 12:529–43. 10.1007/s13679-023-00536-x 37924422 PMC10748777

[26] Batch JT Lamsal SP Adkins M Sultan S Ramirez MN. Advantages and disadvantages of the ketogenic diet: a review article. Cureus. (2020) 12:e9639. 10.7759/cureus.9639 32923239 PMC7480775

[27] Schugar RC Crawford PA. Low-carbohydrate ketogenic diets, glucose homeostasis, and nonalcoholic fatty liver disease. Curr Opin Clin Nutr Metab Care. (2012) 15:374–80. 10.1097/MCO.0b013e3283547157 22617564 PMC3679496

[28] Tummolo A Carella R De Giovanni D Paterno G Simonetti S Tolomeo M. Micronutrient deficiency in inherited metabolic disorders requiring diet regimen: a brief critical review. Int J Mol Sci. (2023) 24:2317024. 10.3390/ijms242317024 38069347 PMC10707160

[29] Portalatin G Shettigar S Carrion-Rodriguez A Medikayala S Herlitz L Sandy D. Ketogenic-diet shake containing *Uncaria tomentosa*-associated acute interstitial nephritis case rep. Nephrol Dial. (2022) 12:219–25. 10.1159/000526391 36465572 PMC9710449

[30] Lian Y Li X Lan Y Li Z Lin X Huang J. Bibliometric and visual analysis in the field of tea in cancer from 2013 to 2023. Front Oncol. (2024) 13:1296511. 10.3389/fonc.2023.1296511 38273848 PMC10808711

[31] Huang J Zhang L Li B Lian Y Lin X Li Z. Bibliometric and visual analysis in the field of two-dimensions nano black phosphorus in cancer from 2015 to 2023. Discov Oncol. (2024) 15:260. 10.1007/s12672-024-01104-y 38961044 PMC11222346

[32] Aria M Cuccurullo C. Bibliometrix: an R-tool for comprehensive science mapping analysis. J Informetr. (2017) 11:959–75. 10.1016/j.joi.2017.08.007

[33] Van Eck N Waltman L. Software survey: VOSviewer, a computer program for bibliometric mapping. Scientometrics. (2010) 84:523–38. 10.1007/s11192-009-0146-3 20585380 PMC2883932

[34] Chen C. CiteSpace II: detecting and visualizing emerging trends and transient patterns in scientific literature. J Am Soc Inform Sci Tech. (2006) 57:359–77. 10.1002/asi.20317

[35] Rafiullah M Musambil M David SK. Effect of a very low-carbohydrate ketogenic diet vs. recommended diets in patients with type 2 diabetes: a meta-analysis. Nutr Rev. (2022) 80:488–502. 10.1093/nutrit/nuab040 34338787

[36] Goldenberg JZ Day A Brinkworth GD Sato J Yamada S Jönsson T. Efficacy and safety of low and very low carbohydrate diets for type 2 diabetes remission: systematic review and meta-analysis of published and unpublished randomized trial data. Br Med J. (2021) 372:m4743. 10.1136/bmj.m4743 33441384 PMC7804828

[37] Saslow LR Daubenmier JJ Moskowitz JT Kim S Murphy EJ Phinney SD. Twelve-month outcomes of a randomized trial of a moderate-carbohydrate versus very low-carbohydrate diet in overweight adults with type 2 diabetes mellitus or prediabetes. Nutr Diabet. (2017) 7:304. 10.1038/s41387-017-0006-9 29269731 PMC5865541

[38] Wei S-J Schell JR Chocron ES Varmazyad M Xu G Chen WH. Ketogenic diet induces p53-dependent cellular senescence in multiple organs. Sci Adv. (2024) 10:eado1463. 10.1126/sciadv.ado1463 38758782 PMC11100565

[39] Barrea L Vetrani C Caprio M Cataldi M Ghoch ME Elce A. From the ketogenic diet to the mediterranean diet: the potential dietary therapy in patients with obesity after COVID-19 infection (post CoVID syndrome). Curr Obes Rep. (2022) 11:144–65. 10.1007/s13679-022-00475-z 35524067 PMC9075143

[40] Gershuni VM Yan SL Medici V. Nutritional ketosis for weight management and reversal of metabolic syndrome. Curr Nutr Rep. (2018) 7:97–106. 10.1007/s13668-018-0235-0 30128963 PMC6472268

[41] Yancy Jr WS Vernon MC Westman EC. A pilot trial of a low-carbohydrate, ketogenic diet in patients with type 2 diabetes. Metab Syndr Relat Disord. (2003) 1:239–43. 10.1089/154041903322716723 18370668

[42] Weykamp C. HbA1c: a review of analytical and clinical aspects. Ann Lab Med. (2013) 33:393. 10.3343/alm.2013.33.6.393 24205486 PMC3819436

[43] Feinman RD Pogozelski WK Astrup A Bernstein RK Fine EJ Westman EC. Dietary carbohydrate restriction as the first approach in diabetes management: critical review and evidence base. Nutrition. (2015) 31:1–13. 10.1016/j.nut.2014.06.011 25287761

[44] Krebs JD Parry Strong A Cresswell P Reynolds AN Hanna A Haeusler S. A randomised trial of the feasibility of a low carbohydrate diet vs. standard carbohydrate counting in adults with type 1 diabetes taking body weight into account. Asia Pac J Clin Nutr. (2016) 25:78–84. 10.6133/apjcn.2016.25.1.11 26965765

[45] Dashti HM Mathew TC Al-Zaid NS. Efficacy of low-carbohydrate ketogenic diet in the treatment of type 2 diabetes. Med Princ Pract. (2021) 30:223–35. 10.1159/000512142 33040057 PMC8280429

[46] Tagliabue A Bertoli S Trentani C Borrelli P Veggiotti P. Effects of the ketogenic diet on nutritional status, resting energy expenditure, and substrate oxidation in patients with medically refractory epilepsy: a 6-month prospective observational study. Clin Nutr. (2012) 31:246–9. 10.1016/j.clnu.2011.09.012 22019282

[47] Stefan N. Causes, consequences, and treatment of metabolically unhealthy fat distribution. Lancet Diabet Endocrinol. (2020) 8:616–27. 10.1016/S2213-8587(20)30110-8 32559477

[48] Bashir B Schofield J Downie P France M Ashcroft DM Wright AK. Beyond LDL-C: unravelling the residual atherosclerotic cardiovascular disease risk landscape-focus on hypertriglyceridaemia. Front Cardiovasc Med. (2024) 11:1389106. 10.3389/fcvm.2024.1389106 39171323 PMC11335737

[49] Georgoulis M Chrysohoou C Georgousopoulou E Damigou E Skoumas I Pitsavos C. Long-term prognostic value of LDL-C, HDL-C, lp(a) and TG levels on cardiovascular disease incidence, by body weight status, dietary habits and lipid-lowering treatment: the ATTICA epidemiological cohort study (2002–2012). Lipids Health Dis. (2022) 21:141. 10.1186/s12944-022-01747-2 36529737 PMC9762061

[50] Yuan X Wang J Yang S Gao M Cao L Li X. Effect of the ketogenic diet on glycemic control, insulin resistance, and lipid metabolism in patients with T2DM: a systematic review and meta-analysis. Nutr Diabetes. (2020) 10:38. 10.1038/s41387-020-00142-z 33257645 PMC7705738

[51] Cahill GF Jr. Fuel metabolism in starvation. Annu Rev Nutr. (2006) 26:1–22. 10.1146/annurev.nutr.26.061505.111258 16848698

[52] Farrés J Pujol A Coma M Ruiz JL Naval J Mas JM. Revealing the molecular relationship between type 2 diabetes and the metabolic changes induced by a very-low-carbohydrate low-fat ketogenic diet. Nutr Metab. (2010) 7:88. 10.1186/1743-7075-7-88 21143928 PMC3009973

[53] Zhao P Tian D Song G Ming Q Liu J Shen J. Neferine promotes GLUT4 expression and fusion with the plasma membrane to induce glucose uptake in L6 cells. Front Pharmacol. (2019) 10:999. 10.3389/fphar.2019.00999 31551792 PMC6737894

[54] Atkinson BJ Griesel BA King CD Josey MA Olson AL. Moderate GLUT4 overexpression improves insulin sensitivity and fasting triglyceridemia in high-fat diet-fed transgenic mice. Diabetes. (2013) 62:2249–58. 10.2337/db12-1146 23474483 PMC3712063

[55] Lehninger AL. Sudduth H, Wise JB. D-β-hydroxybutyric dehydrogenase of mitochondria. J Biol Chem. (1960) 235:2450–5. 10.1016/S0021-9258(18)64641-1 14415394

[56] Shimazu T Hirschey MD Newman J He W Shirakawa K Moan NL. Suppression of oxidative stress by β-hydroxybutyrate, an endogenous histone deacetylase inhibitor. Science. (2013) 339:211–4. 10.1126/science.1227166 23223453 PMC3735349

[57] Gallinari P Di Marco S Jones P Pallaoro M Steinkühler C. HDACs, histone deacetylation and gene transcription: from molecular biology to cancer therapeutics. Cell Res. (2007) 17:195–211. 10.1038/sj.cr.7310149 17325692

[58] Watson N Kuppuswamy S Ledford WL Sukumari-Ramesh S. The role of HDAC3 in inflammation: mechanisms and therapeutic implications. Front Immunol. (2024) 15:1419685. 10.3389/fimmu.2024.1419685 39050859 PMC11266039

[59] Halili MA Andrews MR Labzin LI Schroder K Matthias G Cao C. Differential effects of selective HDAC inhibitors on macrophage inflammatory responses to the Toll-like receptor 4 agonist LPS. J Leukoc Biol. (2010) 87:1103–14. 10.1189/jlb.0509363 20200406

[60] Li X Yang J Zhou X Dai C Kong M Xie L. Ketogenic diet-induced bile acids protect against obesity through reduced calorie absorption. Nat Metab. (2024) 6:1397–414. 10.1038/s42255-024-01072-1 38937659

[61] Luc K Schramm-Luc A Guzik TJ Mikolajczyk TP. Oxidative stress and inflammatory markers in prediabetes and diabetes. J Physiol Pharmacol. (2019) 70:1. 10.26402/jpp.2019.6.01 32084643

[62] Sasso FC Salvatore T Tranchino G Cozzolino D Caruso AA Persico M. Cochlear dysfunction in type 2 diabetes: a complication independent of neuropathy and acute hyperglycemia. Metabolism. (1999) 48:1346–50. 10.1016/S0026-0495(99)90141-5 10582539

[63] Ang QY Alexander M Newman JC Tian Y Cai J Upadhyay V. Ketogenic diets alter the gut microbiome resulting in decreased intestinal Th17 cells. Cell. (2020) 181:1263–75.e16. 10.1016/j.cell.2020.04.027 32437658 PMC7293577

[64] Osler W. The Principles and Practice of Medicine. Boston, MA: D. Appleton (1916).

[65] DAFNE Study Group. Training in flexible, intensive insulin management to enable dietary freedom in people with type 1 diabetes: dose adjustment for normal eating (DAFNE) randomised controlled trial. Br Med J. (2002) 325:746. 10.1136/bmj.325.7367.746 12364302 PMC128375

[66] Lennerz BS Barton A Bernstein RK Dikeman RD Diulus C Hallberg S. Management of type 1 diabetes with a very low-carbohydrate diet. Pediatrics. (2018) 141:e20173349. 10.1542/peds.2017-3349 29735574 PMC6034614

[67] Bernstein RK. Dr. Bernstein's Diabetes Solution: the Complete Guide to Achieving Normal Blood Sugars. Boston, MA: Little, Brown (2011).

[68] McClean AM Montorio L McLaughlin D McGovern S Flanagan N. Can a ketogenic diet be safely used to improve glycaemic control in a child with type 1 diabetes? Arch Dis Child. (2019) 104:501–4. 10.1136/archdischild-2018-314973 30470684

[69] Rydin AA Spiegel G Frohnert BI Kaess A Oswald L Owen D. Medical management of children with type 1 diabetes on low-carbohydrate or ketogenic diets. Pediatr Diabet. (2021) 22:448–54. 10.1111/pedi.13179 33470021 PMC10038004

[70] de Bock M Lobley K Anderson D Davis E Donaghue K Pappas M. Endocrine and metabolic consequences due to restrictive carbohydrate diets in children with type 1 diabetes: an illustrative case series. Pediatr Diabet. (2018) 19:129–37. 10.1111/pedi.12527 28397413

[71] Simm PJ Bicknell-Royle J Lawrie J Nation J Draffin K Stewart KG. The effect of the ketogenic diet on the developing skeleton. Epilepsy Res. (2017) 136:62–6. 10.1016/j.eplepsyres.2017.07.014 28778055

[72] Nielsen JV Gando C Joensson E Paulsson C. Low carbohydrate diet in type 1 diabetes, long-term improvement and adherence: a clinical audit. Diabetol Metab Syndr. (2012) 4:23. 10.1186/1758-5996-4-23 22650646 PMC3583262

[73] Willi SM Martin K Datko FM Brant BP. Treatment of type 2 diabetes in childhood using a very-low-calorie diet. Diabet Care. (2004) 27:348–53. 10.2337/diacare.27.2.348 14747212

[74] Li Z Liao X Qin Y Jiang C Lian Y Lin X. Exploring the impact of coffee consumption on liver health: a comprehensive bibliometric analysis. Heliyon. (2024) 10:4721431. 10.2139/ssrn.4721431 38778998 PMC11108974

[75] Nichols JJ Jones LW Morgan PB Efron N. Bibliometric analysis of the meibomian gland literature. Ocul Surf. (2021) 20:212–4. 10.1016/j.jtos.2021.03.004 33757912

[76] Wu X Shen YS Cui S. Global trends in green space and senior mental health studies: bibliometric review. Int J Environ Res Public Health. (2023) 20:21316. 10.3390/ijerph20021316 36674070 PMC9858913

